# AAEM/ADH Self-Crosslinking Waterborne Polyurethane–Acrylate Binder for High-Adhesion Waterborne Inks on PET Films

**DOI:** 10.3390/polym18192453

**Published:** 2026-10-08

**Authors:** Junqing Xi, Chenglong Jiao, Chao Liu, Shixiong Sun, Benbo Zhao, Yunjun Luo

**Affiliations:** 1 School of Chemistry and Chemical Engineering, North University of China, Taiyuan 030051, China; 2 Dezhou Industrial Technology Research Institute of North University of China, Dezhou 253034, China; 3 School of Materials Science and Technology, Beijing Institute of Technology, Beijing 100081, China

**Keywords:** waterborne polyurethane–acrylate, ketone–hydrazide crosslinking, waterborne ink, self-crosslinking, adhesion

## Abstract

To address the poor adhesion of waterborne inks to polyethylene terephthalate (PET) films, a low-temperature ketone–hydrazide self-crosslinking system based on 2-(acetoacetoxy)ethyl methacrylate (AAEM) and adipic acid dihydrazide (ADH) was incorporated into a waterborne polyurethane–acrylate emulsion. Self-crosslinking waterborne polyurethane–acrylate emulsions (AWPUAs) with different AAEM contents were prepared and subsequently used as binders in waterborne inks for PET films. The results showed that increasing the AAEM content reduced the water absorption of the latex films while increasing their water contact angle and tensile strength. Meanwhile, the adhesion of the resulting waterborne inks to corona-treated PET films increased with increasing AAEM content, together with a substantial enhancement in re-solubility. At an AAEM content of 7 wt%, the AWPUA emulsion exhibited good colloidal stability, and the corresponding waterborne ink achieved nearly 100% adhesion while maintaining good re-solubility. These findings demonstrate that AAEM/ADH ketone–hydrazide self-crosslinking modification provides an effective approach for improving the film properties of waterborne polyurethane–acrylate emulsions and the application performance of waterborne inks on PET films.

## 1. Introduction

The development of green printing and sustainable packaging has driven the transition of printing inks from solvent-borne to waterborne systems [1,2]. Waterborne inks use water as the primary dispersion medium and offer the advantages of low volatile organic compound (VOC) emissions and low odor. Consequently, they have attracted considerable attention in food packaging, plastic film printing, decorative printing [3,4,5,6,7]. However, plastic films, particularly polyethylene terephthalate (PET) films, generally exhibit low surface energy and weak surface polarity. As a result, waterborne inks are prone to insufficient wetting and spreading, cratering defects, and poor interfacial adhesion on these substrates [8,9]. These limitations hinder the broader application of waterborne inks in plastic-film packaging and printing.

The structural design of binder resins is crucial for improving the overall performance of waterborne inks [2,6,7,8,9,10]. Common binders for waterborne inks include waterborne acrylates, waterborne polyurethanes, waterborne epoxy resins, and their hybrid systems. Among these, waterborne polyurethanes exhibit good flexibility owing to their mobile soft segments, while polar urethane groups can promote intermolecular and interfacial interactions, thereby contributing to adhesion to substrates [11,12,13], whereas acrylate resins offer advantages in hardness, weather resistance, and film-forming properties [14]. The hybridization of polyurethane and polyacrylate can, to some extent, combine the complementary properties of the two resin systems [15,16,17,18,19,20,21,22,23,24].

However, their adhesion to low-surface-energy plastic films, such as PET, still requires further improvement. Common modification strategies include silicone modification [25], epoxy modification [26], fluorination modification [27], nanocomposite modification [28], and crosslinking modification [29]. Among these approaches, crosslinking modification enables the formation of a network structure during film formation, thereby increasing film compactness and strengthening interactions between polymer chains. The resulting crosslinked network restricts polymer-chain mobility, increases film compactness and cohesive strength, and reduces the penetration of water molecules. These structural changes contribute to improved mechanical strength, water resistance, thermal stability, and substrate adhesion [30,31,32,33].

Ketone–hydrazide crosslinking systems have attracted considerable attention because of their mild reaction conditions and ability to undergo crosslinking at room temperature (25 ± 2 °C) or relatively low temperatures [34,35,36,37]. For example, Parra et al. [38] prepared self-crosslinking protein–acrylate hybrid emulsions through the DAAM/ADH ketone–hydrazide reaction. During drying and film formation, hydrazone-bonded crosslinked networks were formed, thereby improving the solvent resistance and mechanical properties of the resulting films. Tian et al. [35] developed an acrylic waterborne ink binder for plastic films using an AAA/HDA low-temperature self-crosslinking system. The resulting self-crosslinked structure improved the binder properties and enhanced both the adhesion and drying performance of the waterborne ink on plastic films.

AAEM not only provides carbonyl sites capable of reacting with the hydrazide groups of ADH, but also contains a methacrylate moiety that can participate in free-radical copolymerization with acrylic monomers, thereby facilitating the incorporation of reactive carbonyl groups into the polymer chains. On this basis, an AAEM/ADH ketone–hydrazide self-crosslinking system was introduced into a waterborne polyurethane–acrylate binder. Unlike previously reported self-crosslinking acrylic binders, the present system combines the structural versatility of polyurethane–acrylate hybrids with low-temperature ketone–hydrazide crosslinking. The effects of AAEM content on emulsion stability, film properties, and the adhesion and re-solubility of the resulting waterborne inks on PET films were systematically investigated.

## 2. Materials and Methods

### 2.1. Materials

Polycarbonate diol (PCDL2000, Tech) was supplied by Shandong Bluestar Dongda Co., Ltd. (Zibo, China). Isophorone diisocyanate (IPDI, Tech), 2-hydroxyethyl methacrylate (HEMA, AR), methyl methacrylate (MMA, AR), butyl acrylate (BA, AR), and acrylamide (AAM, AR) were purchased from Shanghai Macklin Biochemical Co., Ltd. (Shanghai China) Castor oil (CO, Tech) was obtained from Guangzhou Chengyi Chemical Co., Ltd. (Guangzhou, China) 2,2-Bis(hydroxymethyl)propionic acid (DMPA, Tech) was supplied by Shenzhen Jintenglong Industrial Co., Ltd.(Shenzhen, China) Triethylamine (TEA, AR) was supplied by Tianjin Zhiyuan Chemical Reagent Co., Ltd. (Tianjin, China). Dibutyltin dilaurate (DBTDL, CP) was purchased from Aladdin Reagent Co., Ltd. (Shanghai, China). 2-(Acetoacetoxy)ethyl methacrylate (AAEM, CP) was purchased from Guangzhou Jixintai New Materials Co., Ltd. (Guangzhou, China), and adipic acid dihydrazide (ADH, CP) was obtained from Guangdong Yuanfeng Chemical Reagent Co., Ltd. (Shaoguan, China) Ammonium persulfate (APS, AR) was supplied by Fuchen (Tianjin) Chemical Reagent Co., Ltd. Sodium bicarbonate (NaHCO_3_, AR) was supplied by Tianjin Hengxing Chemical Reagent Manufacturing Co., Ltd. (Tianjin, China). The waterborne pigment dispersion was purchased from Yingde Kedi Pigment Technology Co., Ltd. (Guangzhou, China) PET films were purchased from Dongguan Chengnuo Plastic Materials Co., Ltd. (Dongguan, China).

### 2.2. Synthesis of Waterborne Polyurethane Emulsion (WPU)

**Figure 1:**
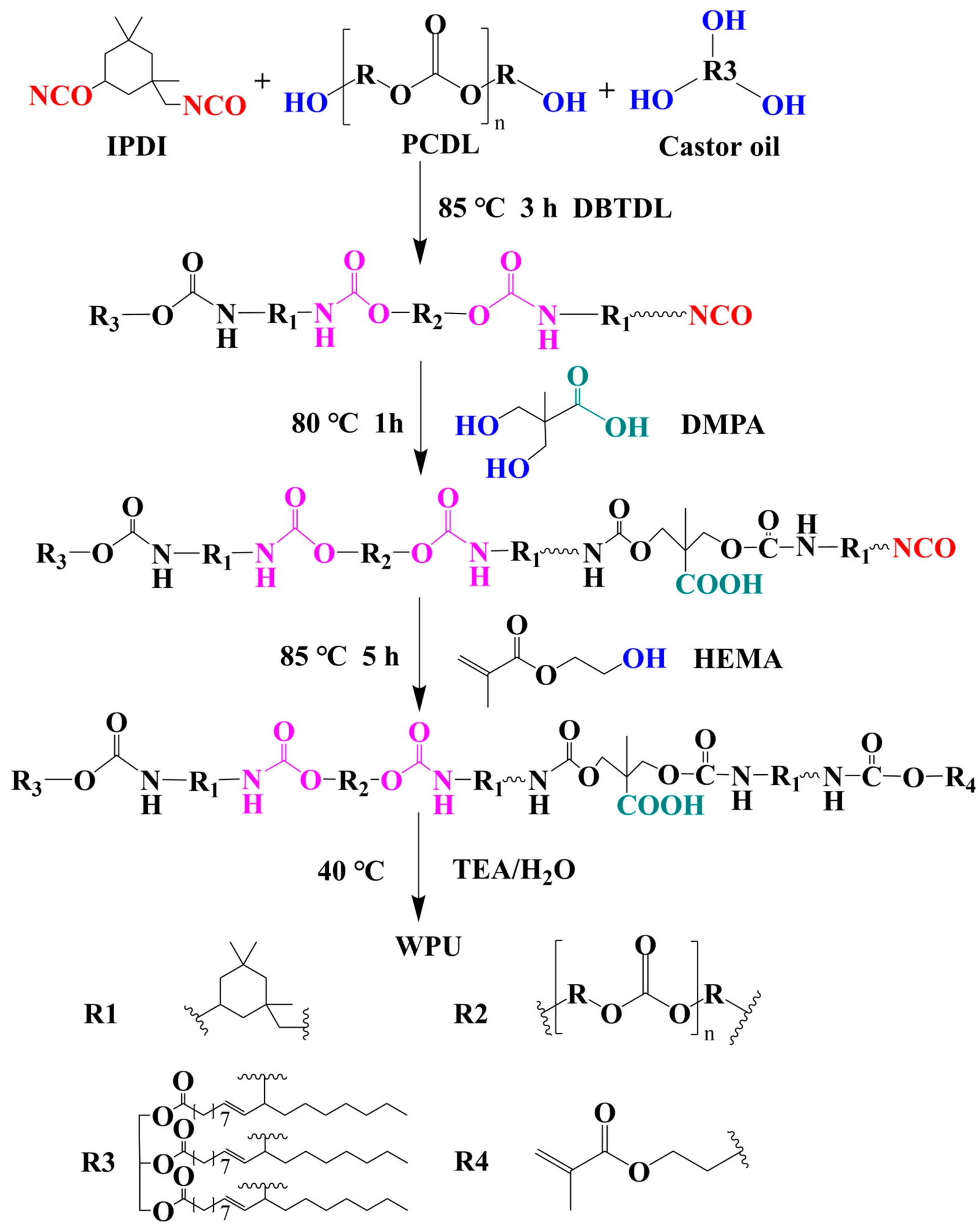
Synthetic route of WPU.

In a typical synthesis, PCDL-2000 (67.7 g), IPDI (34.3 g), CO (21.1 g), and DBTDL (0.001 g) were charged into a four-necked flask equipped with a mechanical stirrer, a thermometer, and a reflux condenser. The mixture was reacted at 85 ± 1 °C for 3 h at a stirring speed of 210 rpm. Subsequently, DMPA (7.0 g) was added, and the reaction was continued at 80 ± 1 °C for 1.5 h. HEMA (9.8 g) was then introduced at 85 ± 1 °C to cap the terminal isocyanate groups, and the reaction was maintained for an additional 5 h. After cooling the reaction mixture to 40 ± 1 °C, a stoichiometric amount of triethylamine (TEA) was added for neutralization, followed by stirring for 0.5 h. Finally, deionized water (255.2 g) was added, and the mixture was subjected to high-speed shear emulsification at 1400 rpm for 1 h to obtain the WPU emulsion. The synthetic route of WPU is illustrated in Figure 1.

### 2.3. Synthesis of Ketone–Hydrazide Self-Crosslinking Waterborne Polyurethane–Acrylate Emulsions (AWPUAs)

**Figure 2:**
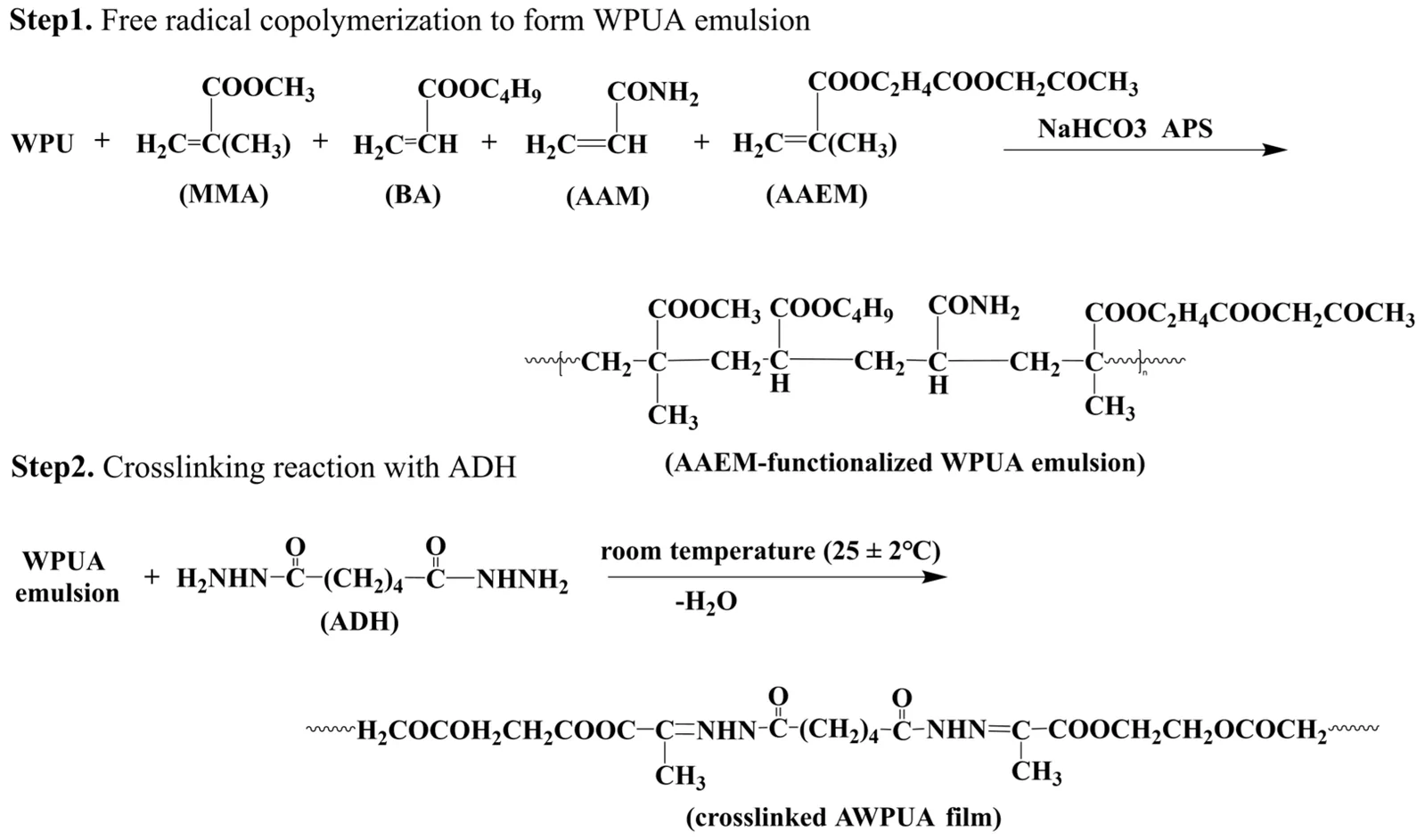
Synthesis route of AWPUA.

Predetermined amounts of MMA, BA, AAM, AAEM, deionized water (130 g), emulsifier (8 g), buffer (0.4 g), and WPU emulsion were mixed thoroughly under high-speed stirring at 800 rpm. One-fifth of the resulting mixture was charged into a four-necked flask equipped with a thermometer, a mechanical stirrer, and a reflux condenser and maintained at 80 ± 1 °C. The initiator (0.2 g) and buffer (0.4 g) were then added to form a bluish seed emulsion. The remaining four-fifths of the emulsion was transferred to a constant-pressure dropping funnel and added dropwise into the flask at a constant rate over 3–5 h. Subsequently, the reaction temperature was raised to 85 ± 1 °C and maintained for 2 h. After an additional amount of initiator (1 g) was added, the temperature was increased to 88 ± 1 °C, and the reaction was continued for another 2 h. The system was then cooled to 50 ± 1 °C, and AMP-95 (7 g) was added to adjust the pH of the emulsion to 8.0, followed by the addition of ADH to obtain the ketone–hydrazide crosslinking-modified WPUA emulsion. The resulting products were designated as AWPUAx, where *x* represents the mass fraction of AAEM relative to the total mass of the acrylic monomers. The reaction route for the synthesis of AWPUA is shown in Figure 2 and the ratio of raw materials is listed in Table 1.

### 2.4. Preparation of Latex Films

An emulsion of 15 ± 2 g was uniformly cast onto a polytetrafluoroethylene (PTFE) plate and allowed to dry naturally at room temperature for 24 h. The film was then dried in a vacuum oven at 50 °C for an additional 24 h to obtain the latex film.

### 2.5. Preparation of Waterborne Ink

**Figure 3:**
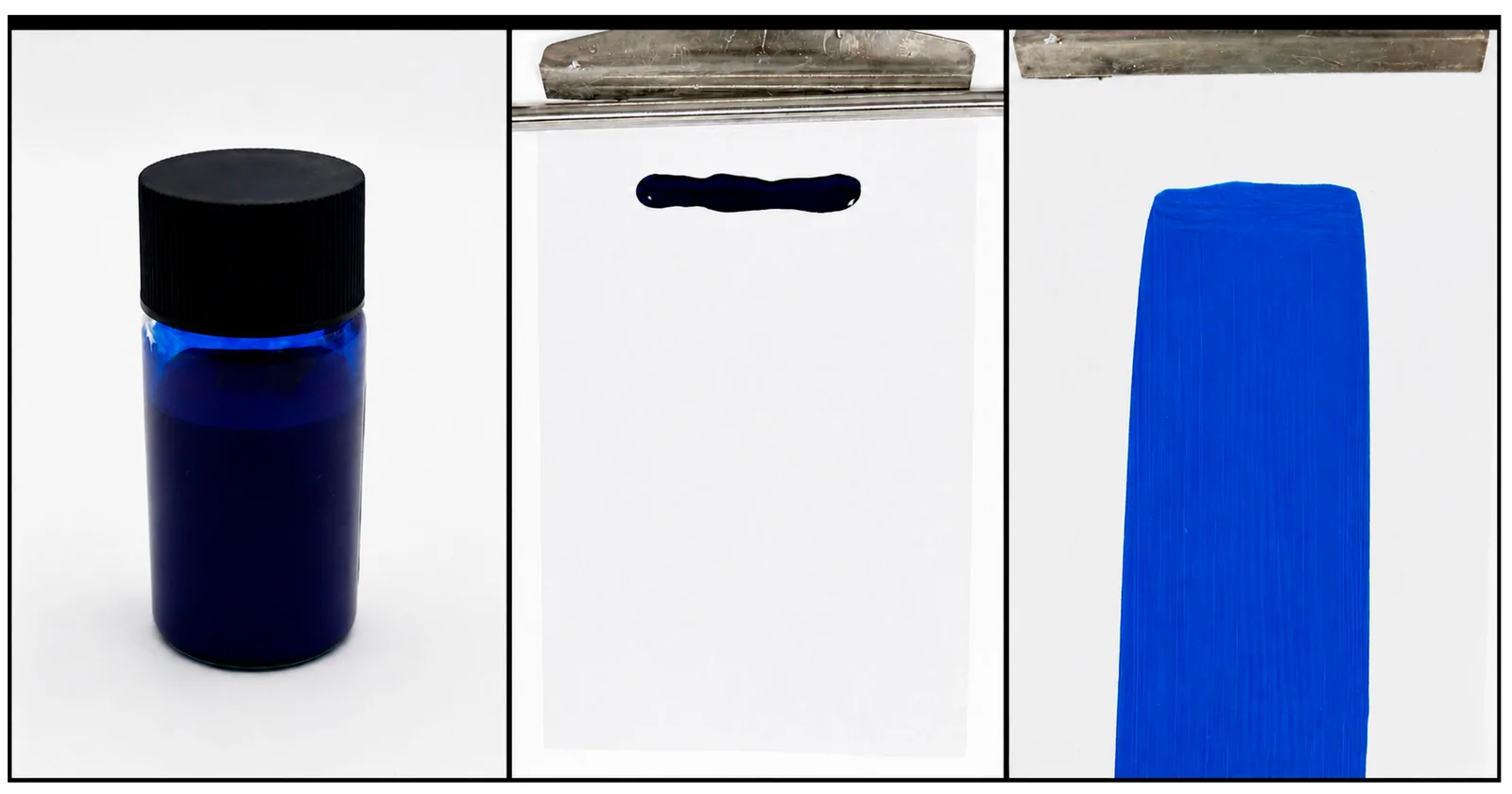
Photographs illustrating the preparation and coating process of the waterborne ink.

The waterborne blue pigment dispersion (4.73 g), waterborne wetting agent (0.05 g), waterborne defoamer (0.02 g), and absolute ethanol (1.2 g) were added to the prepared AWPUA emulsion (24 g) under continuous stirring to obtain the waterborne ink. The resulting ink was then applied onto the commercially corona-treated PET films using a wire-wound bar. The preparation process is illustrated in Figure 3. The coated films were first air-dried at room temperature and subsequently dried in an oven at 50 °C for 24 h.

### 2.6. Characterization

#### 2.6.1. FTIR Analysis

The chemical structures of the WPU, WPUA, and AWPUA films were characterized using a Fourier transform infrared (FTIR) (Nicolet iS50, Thermo Fisher Scientific, Madison, WI, USA) spectrometer in attenuated total reflectance (ATR) mode over a wavenumber range of 4000–400 cm^−1^.

#### 2.6.2. SEM Analysis

The cross-sectional morphology of the AWPUA latex films was examined by scanning electron microscopy (SEM) (MERLIN Compact, Carl Zeiss Microscopy GmbH, Jena, Germany) at an accelerating voltage of 5.0 kV and magnifications of 1000× and 10,000×.

#### 2.6.3. Thermal Stability Test

The thermal decomposition behavior of the films was investigated by thermogravimetric analysis (TGA 3 ^+^) (Mettler-Toledo International, Greifensee, Switzerland) under a nitrogen atmosphere over the temperature range of 25–600 °C at a heating rate of 10 °C min^−1^.

#### 2.6.4. Particle Size and Zeta Potential Measurements

The emulsion was diluted to 0.1 wt% with deionized water, and its average particle size and zeta potential were measured at 25 °C using a nanoparticle size and zeta potential analyzer (Litesizer 500, Anton Paar, Graz, Austria).

#### 2.6.5. Emulsion Viscosity Measurement

The viscosity of the emulsions was measured at 25 °C using an NDJ-79 rotational viscometer (Shanghai Pingxuan Scientific Instrument Co., Ltd., Shanghai, China) equipped with a No.10 spindle. The instrument was operated for 30 s, and the viscosity was recorded after the pointer had stabilized. Each sample was measured in triplicate, and the average value was reported.

#### 2.6.6. Solid Content Measurement

A clean, dry weighing dish was weighed, and its mass was recorded as m_0_. 0.50 ± 0.01 g of the emulsion was added to the weighing dish, and the total mass of the dish and emulsion was recorded as m_1_. The sample was then dried in an oven at 105 °C to a constant mass. After cooling to room temperature in a desiccator, the sample was weighed again, and the resulting mass was recorded as m_2_. The solid content of the emulsion was calculated according to Equation (1):

(1) $$\text{Solid content}\;=\;\frac{m_{2}-m_{0}}{m_{2}-m_{0}}\;\times \;100\%$$

#### 2.6.7. Water Absorption Measurement

At room temperature, the dried resin films were cut into specimens measuring 20 mm × 20 mm × 1 mm and immersed in deionized water for 24 h. The water absorption was calculated according to Equation (2):

(2) $$\text{Water absorption}\;=\;\frac{W-W_{0}}{W_{0}}\;\times \;100\%$$

where W_0_ and W represent the initial and final masses of the latex film, respectively.

#### 2.6.8. Water Contact Angle Measurement

The water contact angles of the AWPUA latex films were measured using a contact angle goniometer(SL-200, Shanghai Solon Information Technology Co., Ltd., Shanghai, China). Each sample was measured three times, and the average water contact angle was calculated.

#### 2.6.9. Mechanical Properties Testing

Tensile tests were performed using a universal testing machine (68TM-30, INSTRON, Norwood, MA, USA). The samples were cut into dumbbell-shaped specimens and subjected to stress–strain testing at room temperature at a crosshead speed of 50 mm min^−1^. Three replicate measurements were conducted for each sample.

#### 2.6.10. Adhesion Test

The waterborne ink was uniformly applied to the surface of corona-treated PET films using a wire-wound bar. The coated films were initially dried at room temperature until a continuous surface film had formed and were subsequently dried in an oven at 50 °C for 24 h. The adhesion of the waterborne ink to the substrate was evaluated using 3M adhesive tape. Adhesion was determined from the area of the ink film removed by the tape, and each sample was tested in triplicate. The area covered by the 3M tape was denoted as (A_0_), while the area of the ink film remaining after tape peeling was denoted as (A).

(3) $$\text{Ink-film retention}\;(\%)\;=\;\frac{A}{A_{0}}\;\times \;100\%$$

#### 2.6.11. Re-Solubility Test

The re-solubility of the AWPUA-based waterborne inks was evaluated using a fineness-of-grind gauge (0–100 μm, Tianjin Jingke Lian Testing Machine Co., Ltd., Tianjin, China). After the gauge was thoroughly cleaned and dried, an appropriate amount of ink was placed at the deep end of the groove and drawn uniformly along the groove using a scraper to form an even ink layer. The coated gauge was then dried at room temperature for 120 s. After drying, the ink layer was rinsed with a 50% aqueous ethanol solution, and the ink residue, streak distribution, and localized residual deposits on the gauge surface were visually examined.

## 3. Results

### 3.1. ATR-FTIR Analysis

**Figure 4:**
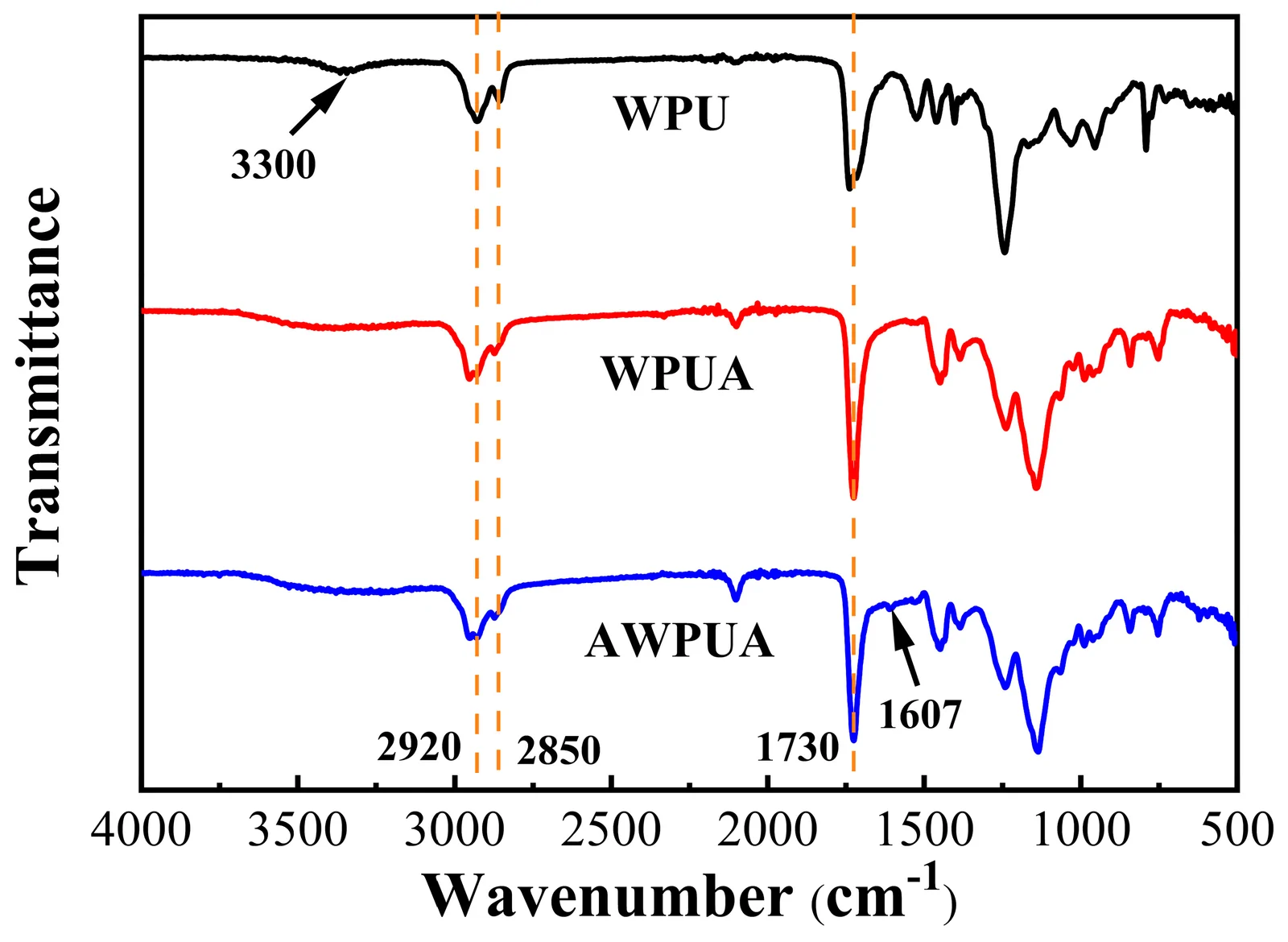
FTIR Spectra of WPU, WPUA, and AWPUA.

The ATR-FTIR spectra of WPU, WPUA, and AWPUA are shown in Figure 4. The absorption bands at approximately 3300, 2920 and 2850, and 1730 cm^−1^ are assigned to N–H stretching, aliphatic C–H stretching, and C=O stretching vibrations, respectively [39]. In addition, no characteristic absorption band of –NCO was observed near 2270 cm^−1^, indicating that the isocyanate groups had been almost completely consumed. The C=C stretching vibration of acrylic monomers is generally observed near 1640 cm^−1^ [40]. However, no distinct C=C absorption band was detected in the spectra of WPUA and AWPUA, indicating that the acrylic monomers underwent free-radical polymerization. Compared with WPU, WPUA and AWPUA exhibited a markedly enhanced C=O absorption band in the range of 1700–1730 cm^−1^, which was attributed to the increased content of ester and carboxyl groups introduced by the acrylic monomers [14,24]. Compared with WPUA, AWPUA exhibited a new absorption band near 1607 cm^−1^, which may be assigned to the C=N stretching vibration of hydrazone bonds, suggesting the occurrence of the ketone–hydrazide reaction between AAEM and ADH [34,35,36,37,38]. Overall, the ATR-FTIR results preliminarily confirmed the successful synthesis of the WPU, WPUA, and AWPUA resins.

### 3.2. Cross-Sectional Morphology of the Latex Films

**Figure 5:**
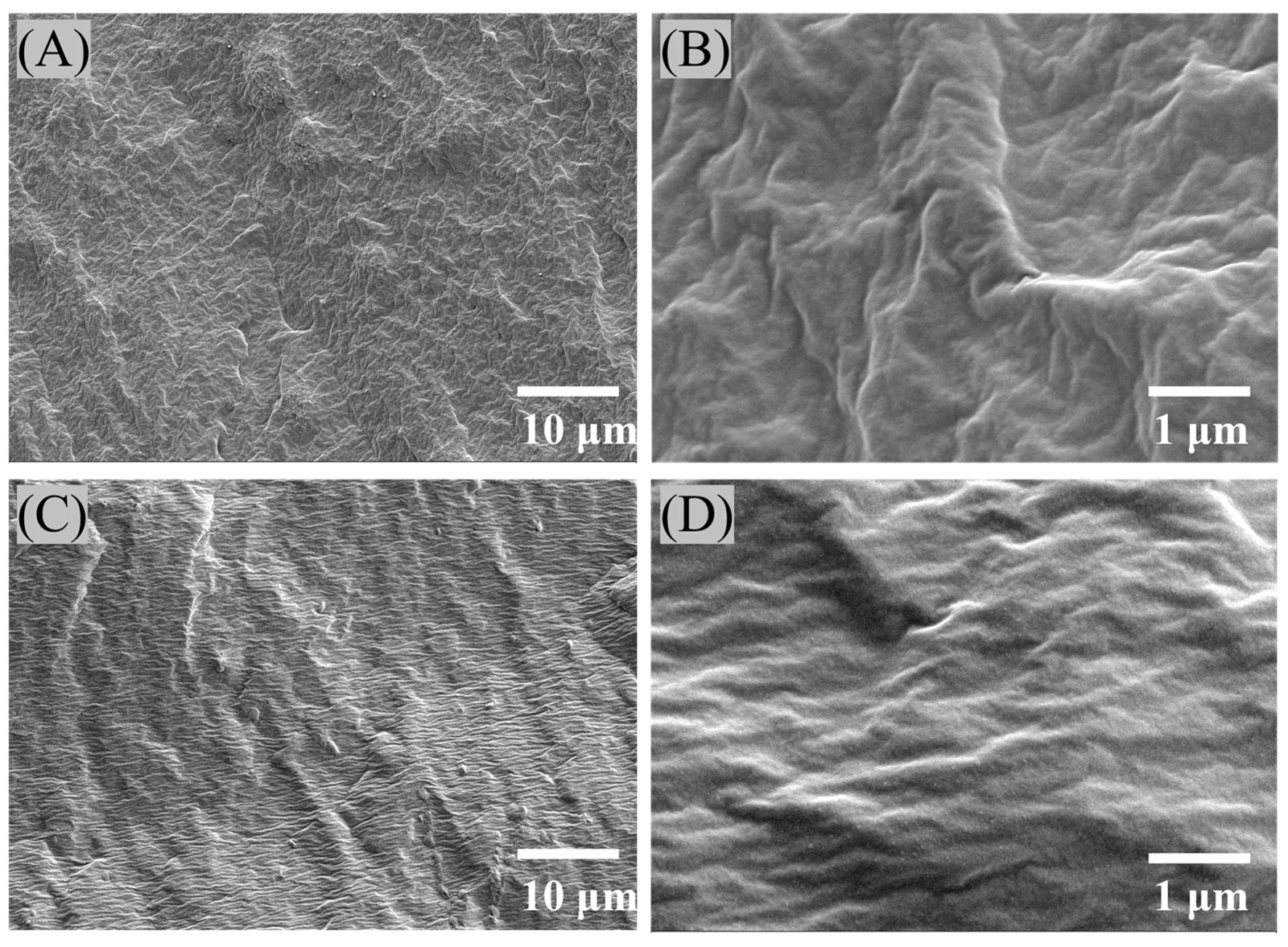
Cross-sectional SEM images of AWPUA latex films obtained at an accelerating voltage of 5.0 kV: AWPUA0 at 1000× (**A**) and 10,000× (**B**), and AWPUA7 at 1000× (**C**) and 10,000× (**D**).

Figure 5 shows the cross-sectional SEM images of the AWPUA0 and AWPUA7 latex films at different magnifications. Figure 5A,B correspond to AWPUA0, while Figure 5C,D correspond to AWPUA7. As shown in Figure 5, the cross section of the AWPUA0 latex film exhibits pronounced irregular wrinkles and localized undulations. In comparison, the AWPUA7 latex film displays a more continuous, layered wrinkled structure with a more uniform overall texture, and no obvious large pores, cracks, or delamination are observed. These results indicate that the incorporation of an appropriate amount of AAEM/ADH contributes to improved film continuity and structural compactness. This may be attributed to the enhanced interactions between polymer chains and the formation of ketone–hydrazide/hydrazone structures during film formation, thereby increasing the cohesive strength and structural integrity of the film [34,35,36,37,38].

### 3.3. Analysis of Basic Emulsion Properties

**Figure 6:**
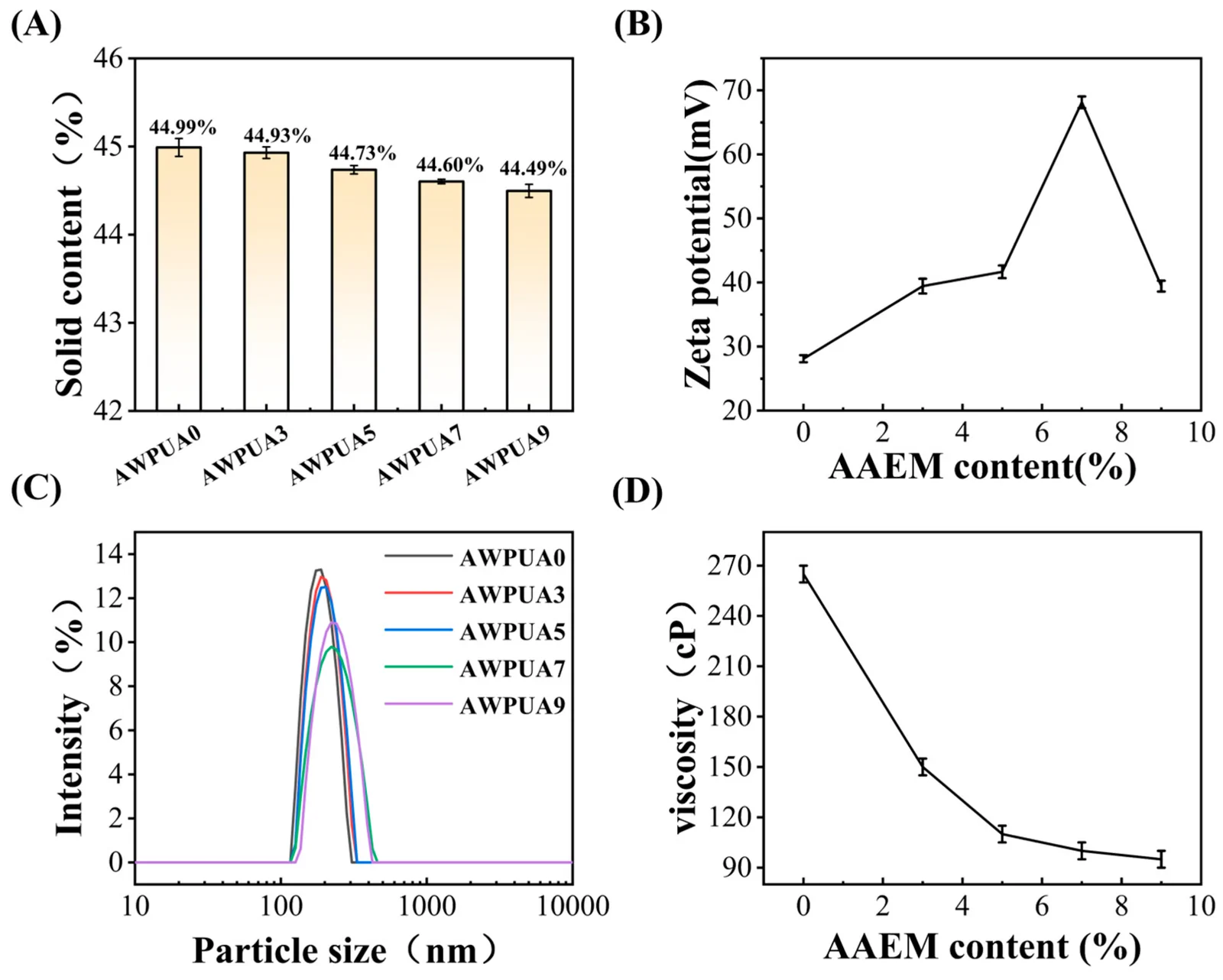
Effects of AAEM content on the properties of AWPUA emulsions: (**A**) solid content; (**B**) zeta potential; (**C**) particle size distribution; and (**D**) viscosity.

Figure 6 shows the effects of AAEM content on the basic properties of the AWPUA emulsions. As shown in Figure 6A, the solid content decreased from 44.99% for AWPUA0 to 44.49% for AWPUA9. The relatively stable solid content minimizes the influence of differences in resin content on the subsequent performance evaluations. Figure 6B shows that the absolute value of the zeta potential initially increased and then decreased with increasing AAEM content. The increase in the absolute zeta potential indicates a higher surface charge density of the latex particles, stronger electrostatic repulsion between particles, and improved colloidal stability of the emulsion [24]. When the AAEM content was further increased to 9 wt%, the absolute zeta potential decreased markedly. This attributed to changes in the hydrophilic–hydrophobic balance and surface-chain arrangement of the latex particles caused by excessive AAEM, which reduced the effective exposure of ionic groups on the particle surfaces and consequently lowered the surface charge density [35,37]. As shown in Figure 6C, the average particle size increased from 198 to 243 nm. The relatively narrow particle size distributions and the absence of distinct aggregation peaks at larger particle sizes indicate good dispersion stability of the emulsions. The increase in particle size was mainly attributed to the introduction of the hydrophobic methacrylate structure of AAEM, which enhanced the hydrophobicity of the polymer chains and promoted their aggregation within the latex particles, thereby increasing the average particle size [35]. Figure 6D shows that the emulsion viscosity decreased from 262 to 96 cP as the AAEM content increased from 0 to 9%. At a nearly constant solid content, the incorporation of AAEM increased the latex-particle size. Larger particles reduce the number of particles per unit volume and the frequency of interparticle contact, thereby decreasing interparticle friction and lowering the emulsion viscosity [23]. Overall, the incorporation of an appropriate amount of AAEM improved the dispersion and electrostatic stability of the AWPUA emulsions. At an AAEM content of 7 wt%, the emulsion exhibited an absolute zeta potential of 68.2 mV, indicating good colloidal stability.

### 3.4. Thermal Stability Analysis of the Latex Films

**Figure 7:**
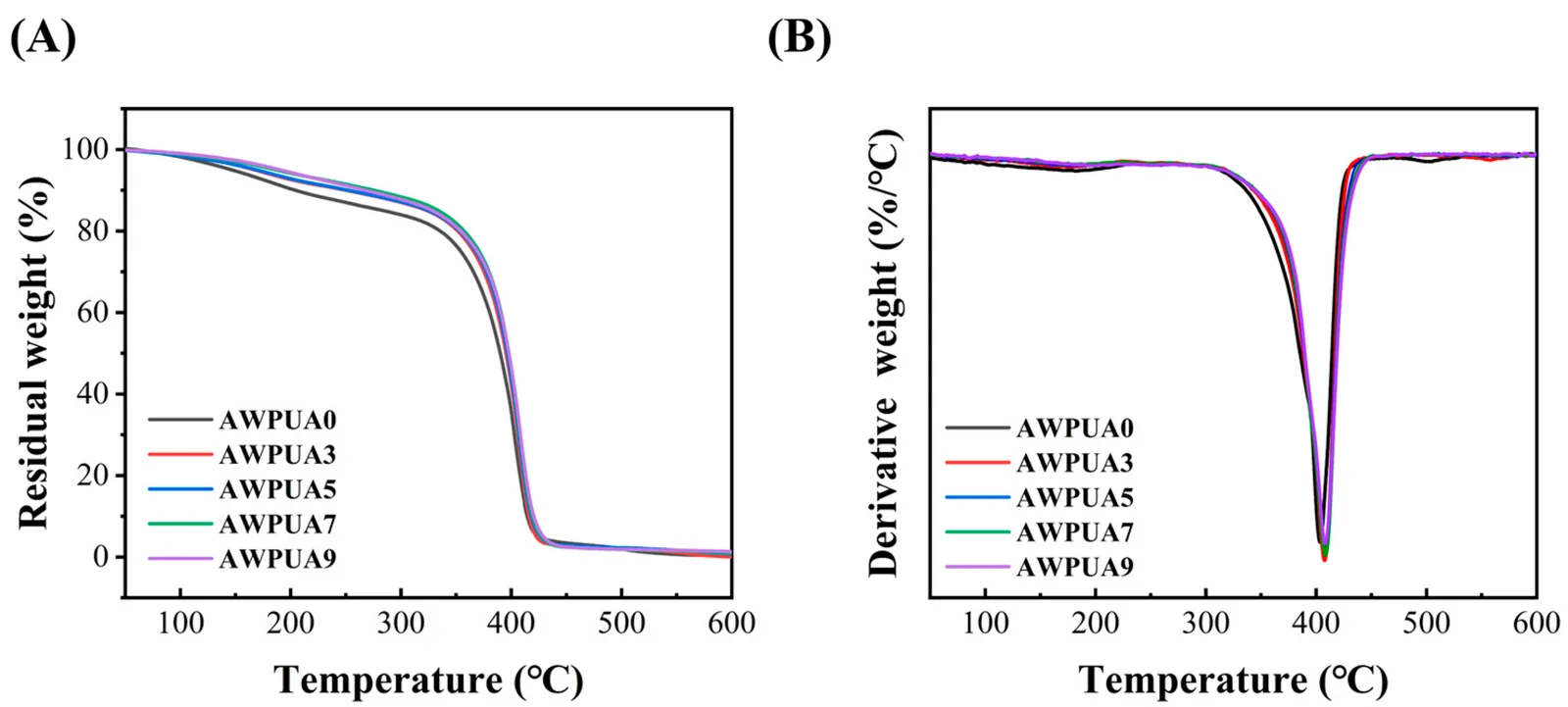
TGA (**A**) and DTG (**B**) curves of different AWPUA samples.

Figure 7 shows the TGA and DTG curves of AWPUA latex films with different AAEM contents. As shown in Figure 7, all samples exhibit similar mass-loss profiles, indicating comparable thermal decomposition processes. Only slight mass loss occurs in the range of 100–300 °C, which is mainly attributed to the evaporation or decomposition of residual moisture, low-molecular-weight additives, and thermally less stable chain segments. Rapid mass loss is observed between 350 and 430 °C, corresponding primarily to the thermal decomposition of the polyurethane segments, polyacrylate segments, and polymer backbones [10,16,18]. The DTG curves show that all samples exhibit a maximum mass-loss-rate peak near 400 °C, indicating that the principal thermal decomposition of the AWPUA latex films is concentrated within this temperature region.

### 3.5. Water Contact Angle and Water Absorption of the Latex Films

**Figure 8:**
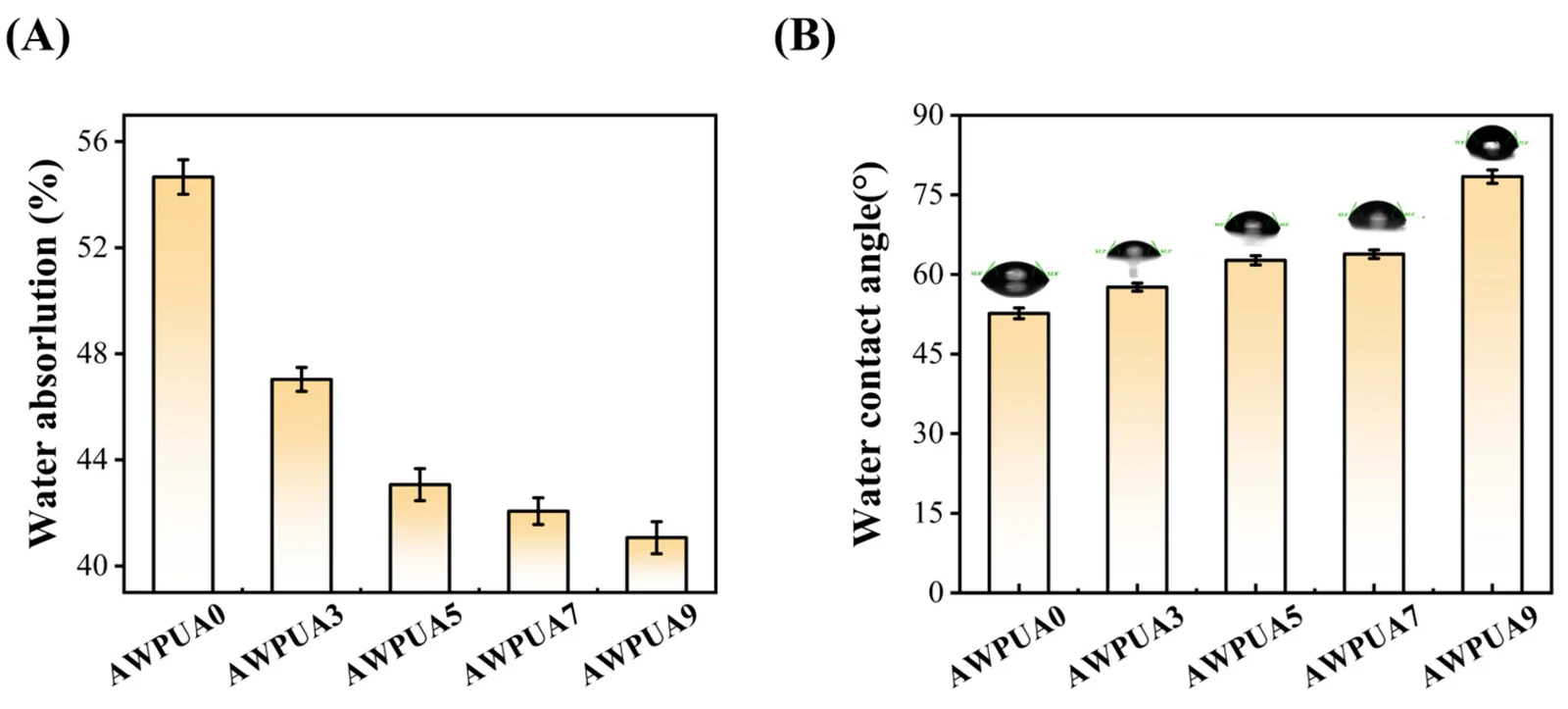
Water resistance of different AWPUA latex films: (**A**) water absorption and (**B**) water contact angle.

Figure 8 shows the effects of AAEM content on the water contact angle and water absorption of the AWPUA latex films. As the AAEM content increased, the water absorption decreased from 55 to 41%, while the water contact angle increased from 53 to 78°, indicating marked improvements in the water resistance and surface hydrophobicity of the films. This behavior was mainly attributed to the increased proportion of hydrophobic structures in the polymer chains following the incorporation of AAEM. In addition, the ketone–hydrazide crosslinked structures formed between AAEM and ADH during film formation, together with hydrogen-bonding interactions, enhanced the compactness of the films. Therefore, the incorporation of AAEM effectively improved the water resistance of the AWPUA latex films [30,31,32,33].

### 3.6. Mechanical Properties Analysis

**Figure 9:**
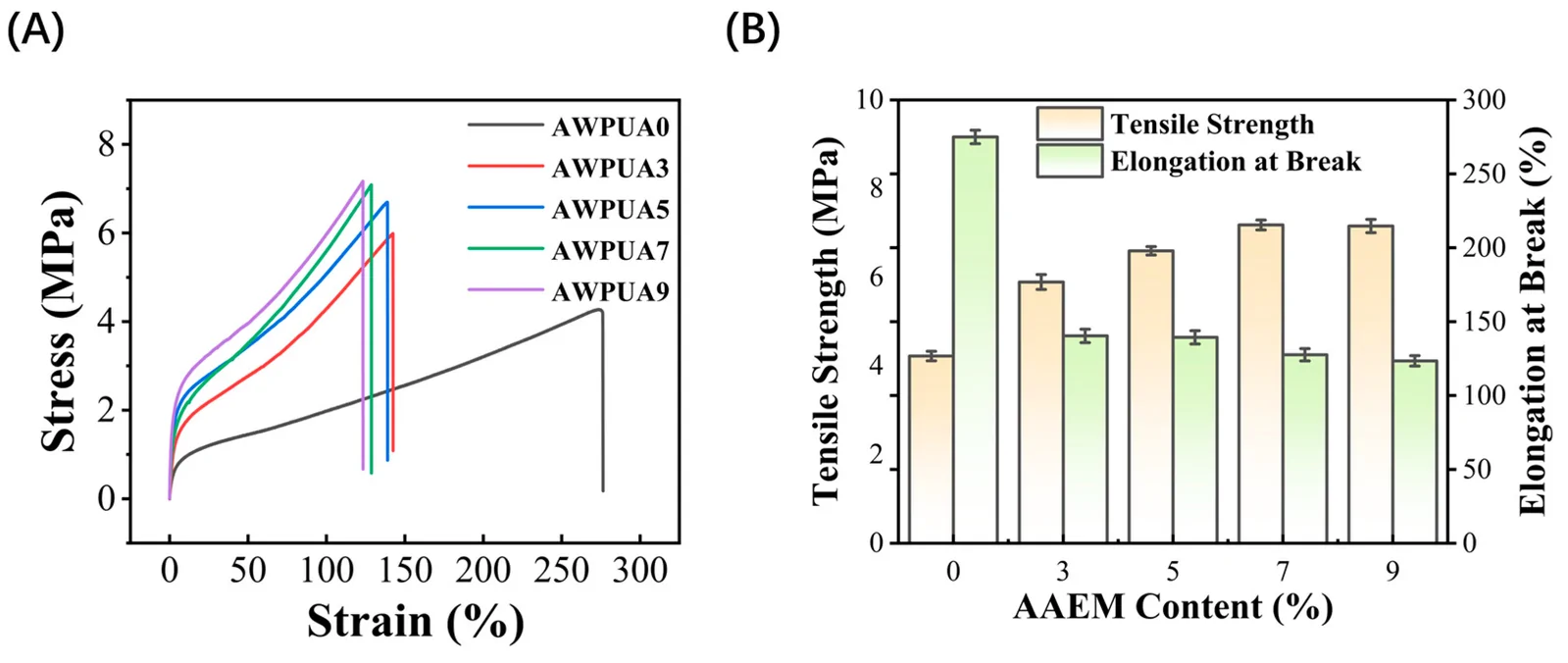
(**A**) Stress–strain curves of different AWPUA samples; (**B**) Tensile strength and elongation at break of different AWPUA samples.

Figure 9 shows the mechanical properties of AWPUA latex films with different AAEM contents. As the AAEM content increased, the maximum tensile strength of the latex films increased from 4.2 to 7.2 MPa, while the elongation at break decreased from 260 to 120%. These results indicate that the incorporation of AAEM/ADH markedly enhanced the film strength while increasing its rigidity. This behavior can be attributed to the formation of hydrazone bonds between the β-ketoester groups of AAEM and the hydrazide groups of ADH [34,35,36,37,38]. Meanwhile, hydrogen-bonding and polar interactions among the hydrazide, urethane, and carboxyl groups were strengthened [31], thereby increasing the intermolecular interactions between polymer chains and producing a denser network structure, which contributed to the improved tensile strength [29,30,31,32,33]. However, the enhanced intermolecular interactions and network structure restricted polymer-chain mobility, resulting in a decrease in elongation at break and an increase in film rigidity. When the AAEM content exceeded 7 wt%, the increase in tensile strength became less pronounced, whereas the elongation at break continued to decrease, indicating that excessive AAEM provided only a limited further improvement in strength.

### 3.7. Tape-Peel Adhesion Test

**Figure 10:**
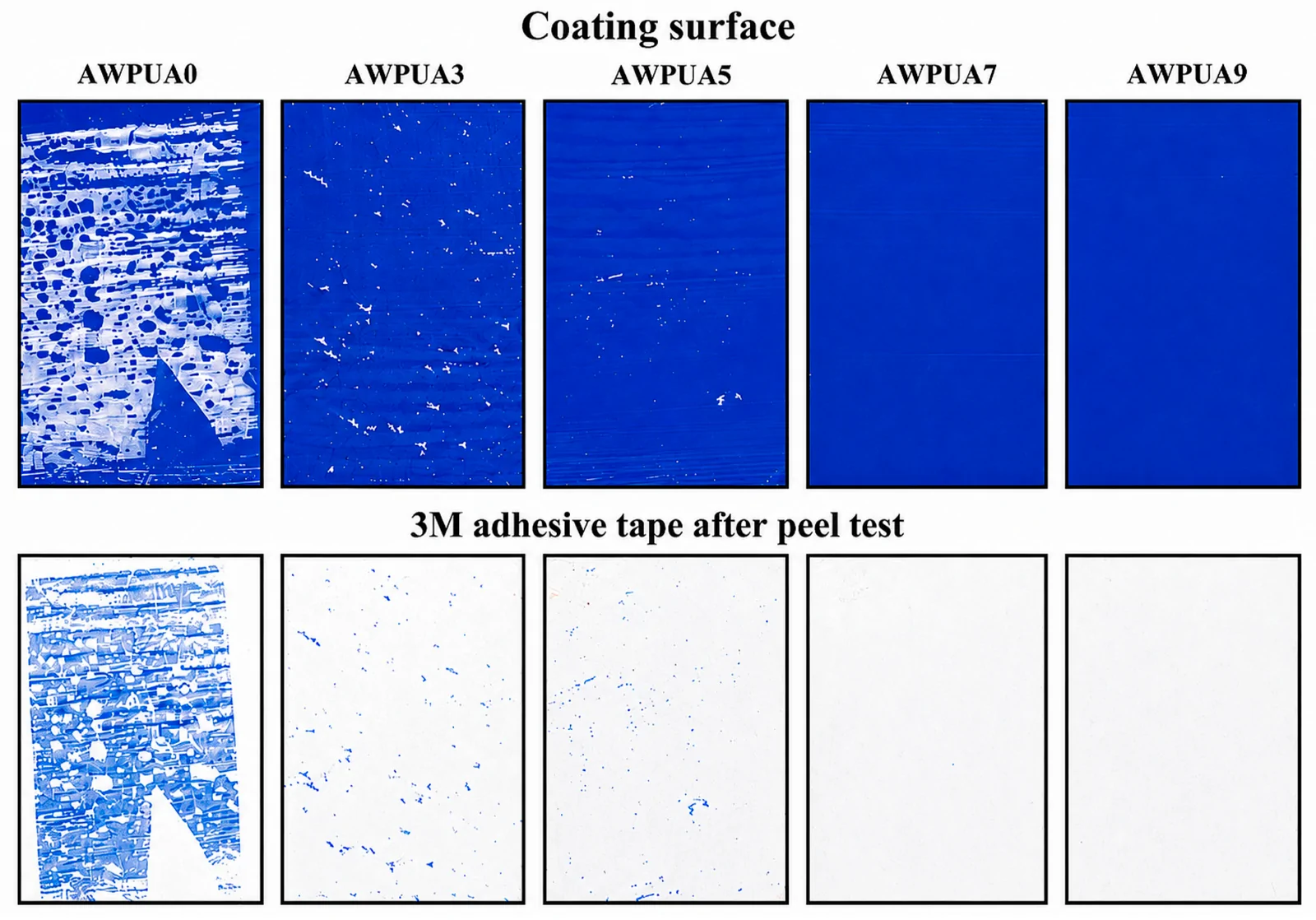
Results of the 3M tape peel test.

As shown in Figure 10 and Table 2, the adhesion of the ink to the PET film increased markedly with increasing AAEM content. The AWPUA0 ink without AAEM exhibited an ink-film retention of 30% after tape peeling, indicating relatively weak interfacial bonding with the PET substrate. When the AAEM content was increased to 3 and 5 wt%, the ink-film retention increased to 80% and 90%, respectively. At AAEM contents of 7 and 9 wt%, the ink-film retention 100%, indicating that the ink film remained essentially intact after tape peeling. These results demonstrate that the incorporation of AAEM significantly improves the adhesion of AWPUA-based waterborne inks to PET films. In particular, the ink containing 7 wt% AAEM already exhibited nearly complete adhesion.

### 3.8. Re-Solubility Analysis of the Waterborne Inks

**Figure 11:**
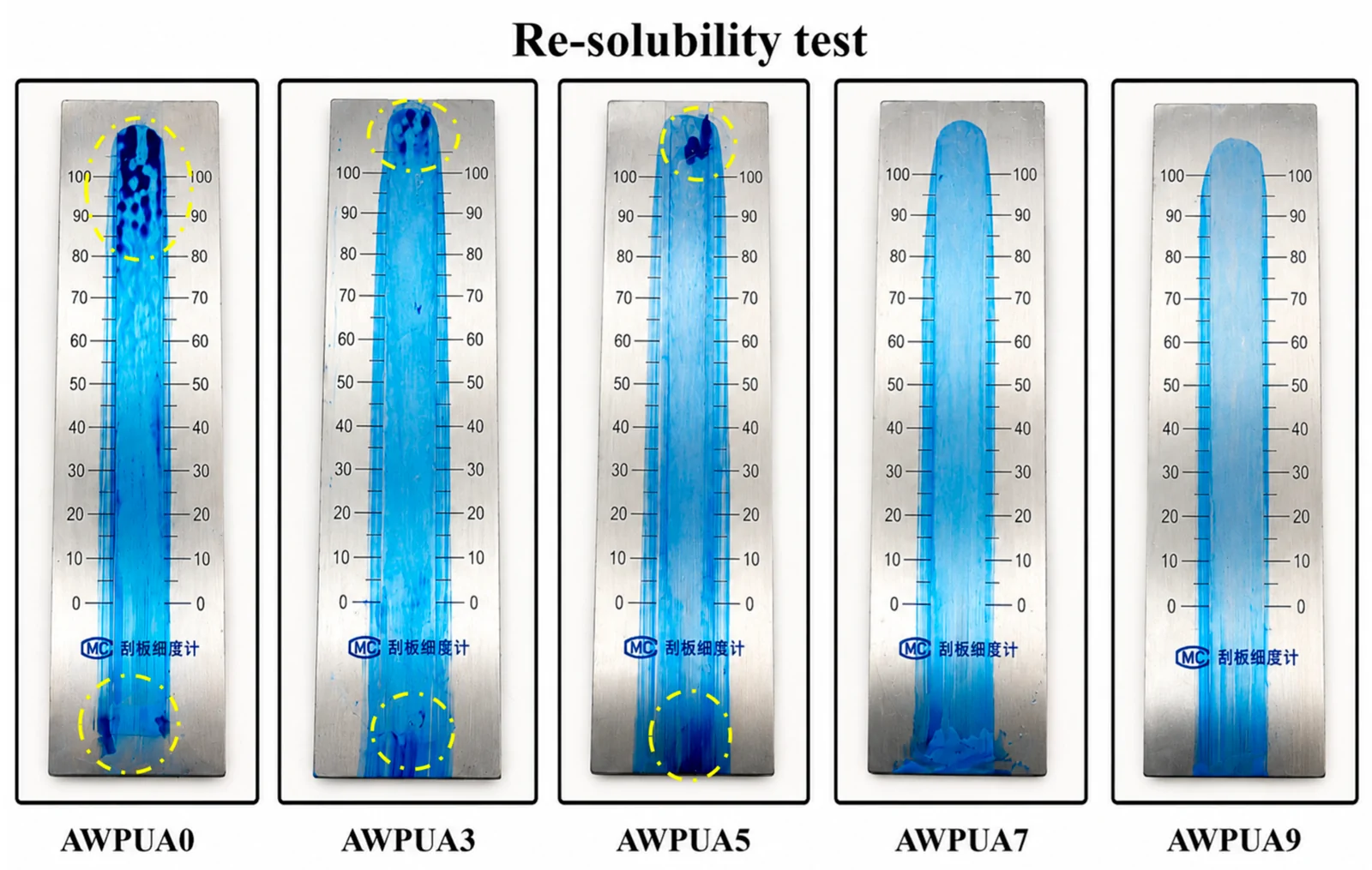
Re-solubility test results of different AWPUA-based waterborne inks. The yellow dashed circles indicate regions with visible residual ink deposits or incompletely redispersed aggregates after the re-solubility test.

Figure 11 shows the re-solubility test results of AWPUA-based waterborne inks with different AAEM contents. As shown in Figure 11, AWPUA0 exhibited obvious ink residues and uneven streaks after re-solubilization, indicating a relatively weak ability of the dried ink layer to be rewetted and redispersed. With increasing AAEM content, the amount of ink residue on the grind gauge decreased and its distribution became more uniform, indicating that the incorporation of an appropriate amount of AAEM/ADH improved the re-solubility of the ink. This decrease is attributed to the enhanced film-forming uniformity of the binder and the improved stability of pigment encapsulation provided by AAEM/ADH, which reduced the formation of pronounced residues during re-solubilization [37].

### 3.9. Mechanistic Investigation

**Figure 12:**
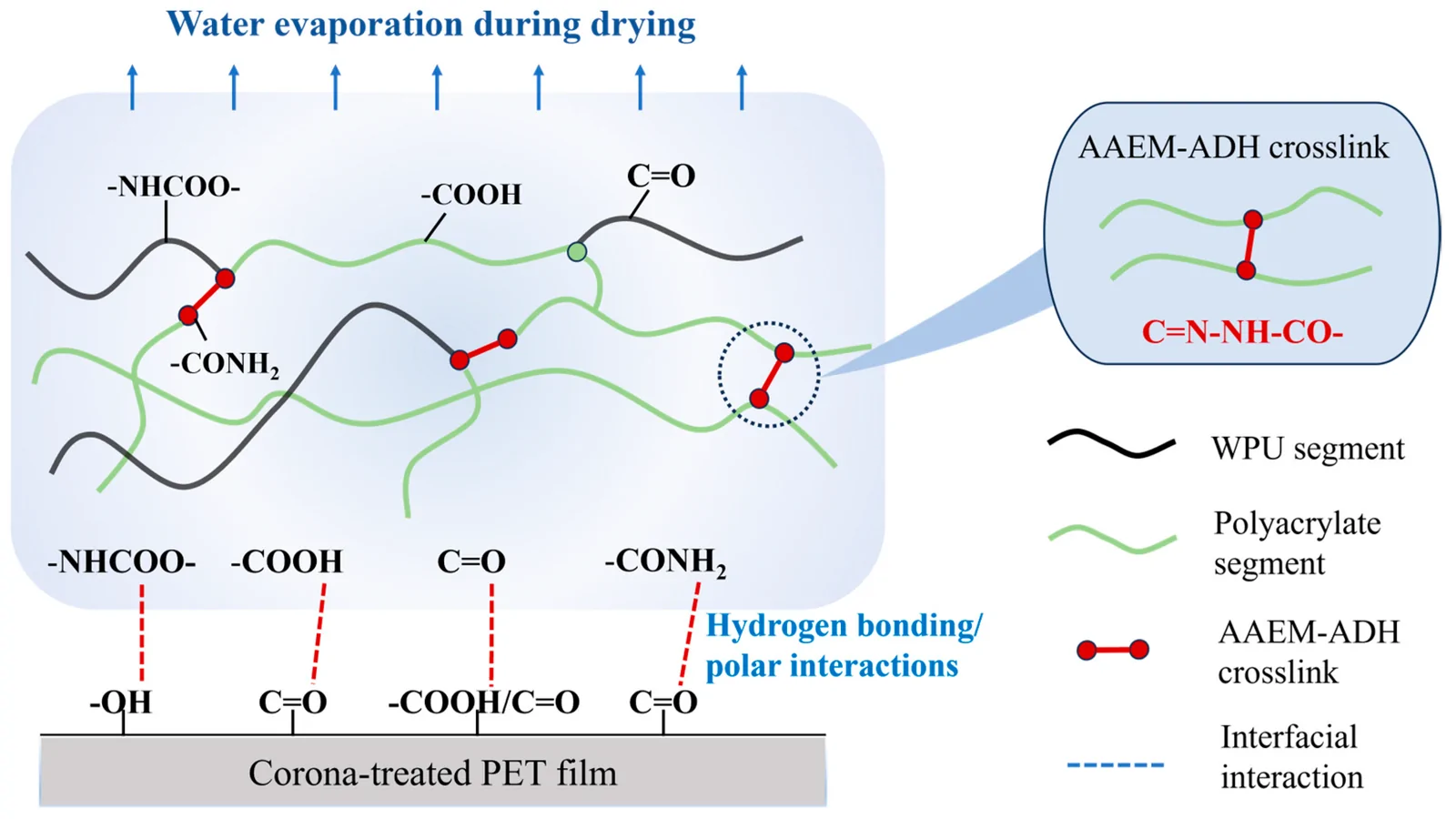
Schematic illustration of AAEM/ADH self-crosslinking during film formation and the interfacial interaction mechanism between AWPUA and corona-treated PET films.

The film-formation and interfacial adhesion mechanisms of the AWPUA-based waterborne ink are illustrated in Figure 12. During drying, evaporation of water causes the latex particles to pack closely, deform, and coalesce, ultimately forming a continuous film. Meanwhile, the β-ketoester groups in the AAEM side chains undergo condensation reactions with the hydrazide groups of ADH to form acylhydrazone bonds characterized by –C=N–NH–CO– linkages. ADH further connects different polymer chains, thereby constructing a covalently crosslinked network [34,35,36,37]. This network acts synergistically with hydrogen-bonding and polar interactions involving urethane, amide, carboxyl, and carbonyl groups, thereby increasing the cohesive strength and structural compactness of the film while restricting the relative motion of the polymer chains.

At the interface between the ink film and the corona-treated PET substrate, the N–H and O–H groups in AWPUA can form N–H···O=C and O–H···O=C hydrogen bonds with carbonyl, carboxyl, and other oxygen-containing groups on the PET surface. In addition, ester, urethane, and carbonyl groups within the system can participate in dipole–dipole interactions, thereby enhancing interfacial adhesion. The combined enhancement of the internal crosslinked network and the ink-film/PET interfacial interactions ultimately improves the overall performance of the ink.

## 4. Conclusions

In this study, a low-temperature AAEM/ADH ketone–hydrazide self-crosslinking system was incorporated into a waterborne polyurethane–acrylate emulsion to prepare AWPUA emulsions with low-temperature self-crosslinking capability, which were subsequently applied as binders in waterborne inks for PET films. With increasing AAEM content, the water resistance and tensile strength of the latex films were improved to varying degrees. In particular, the water absorption decreased from 55 to 41%, while the water contact angle increased from 53 to 78°, indicating increased film hydrophobicity and resistance to water penetration. Meanwhile, the incorporation of AAEM/ADH increased the cohesive strength of the ink film and strengthened its interfacial adhesion to corona-treated PET films, resulting in a substantial improvement in ink adhesion. At an AAEM content of 7 wt%, the AWPUA emulsion exhibited good colloidal stability, and the corresponding waterborne ink achieved nearly 100% adhesion to PET films while maintaining good re-solubility. These results demonstrate that AAEM/ADH ketone–hydrazide self-crosslinking modification can effectively improve the overall performance of waterborne polyurethane–acrylate emulsions and provides a feasible approach for preparing high-adhesion waterborne ink binders for PET films.

## Figures and Tables

**Table 1:** The experimental formulation of AWPUA.

Sample	WPU	MMA	BA	AAM	AAEM		ADH
g	g	g	g	g	ω_AAEM_/%	g	g
AWPUA0	42	77	65	0.7	0	0	0
AWPUA3	42	74	63	0.7	3	4.5	1.5
AWPUA5	42	72	61	0.7	5	7.3	2.4
AWPUA7	42	70	60	0.7	7	10	3.2
AWPUA9	42	69	58	0.7	9	12.5	4.1

**Table 2:** Ink-film retention of AWPUAx-based waterborne inks on corona-treated PET films.

Sample	AAEM Content	Ink-Film Retention (%)
AWPUA0	0%	26
AWPUA3	3%	82
AWPUA5	5%	85
AWPUA7	7%	100
AWPUA9	9%	100

## Data Availability

The original contributions presented in this study are included in the article. Further inquiries can be directed to the corresponding authors.
